# Type-2 Diabetics Reduces Spatial Variation of Microbiome Based on Extracellur Vesicles from Gut Microbes across Human Body

**DOI:** 10.1038/s41598-019-56662-x

**Published:** 2019-12-27

**Authors:** Geumkyung Nah, Sang-Cheol Park, Kangjin Kim, Sungmin Kim, Jaehyun Park, Sanghun Lee, Sungho Won

**Affiliations:** 1 Interdisciplinary Program in Bioinformatics, Seoul National University, Seoul, South Korea; 2 Department of public health sciences, Seoul National university, Seoul, South Korea; 3 Inje University College of Medicine and Haeundae Paik Hospital, Busan, South Korea; 4 Department of medical consilience, Graduate school, Dankook university, Yongin-si, South Korea; 5 Institute of Health and Environment, Seoul National University, Seoul, South Korea

**Keywords:** Microbiome, Calcium and phosphate metabolic disorders

## Abstract

As a result of advances in sequencing technology, the role of gut microbiota in the mechanism of type-2 diabetes mellitus (T2DM) has been revealed. Studies showing wide distribution of microbiome throughout the human body, even in the blood, have motivated the investigation of the dynamics in gut microbiota across the humans. Particularly, extracellular vesicles (EVs), lipid bilayer structures secreted from the gut microbiota, have recently come into the spotlight because gut microbe-derived EVs affect glucose metabolism by inducing insulin resistance. Recently, intestine hyper-permeability linked to T2DM has also been associated with the interaction between gut microbes and leaky gut epithelium, which increases the uptake of macromolecules like lipopolysaccharide from the membranes of microbes leading to chronic inflammation. In this article, we firstly investigate the co-occurrence of stool microbes and microbe-derived EVs across serum and urine in human subjects (N = 284), showing the dynamics and stability of gut derived EVs. Stool EVs are intermediate, while the bacterial composition in both urine and serum EVs is distinct from the stool microbiome. The co-occurrence of microbes was compared between patients with T2DM (N = 29) and matched in healthy subjects (N = 145). Our results showed significantly higher correlations in patients with T2DM compared to healthy subjects across stool, serum, and urine, which could be interpreted as the dysfunction of intestinal permeability in T2DM. Therefore, the significant correlation of EVs might give insight into the pathophysiological mechanisms of T2DM, as well as the role of EVs as a biomarker in the intestinal permeability of T2DM.

## Introduction

Recent advances in the culture-independent investigation of microbial communities, such as sequencing technologies and the development of bioinformatics tools, have revealed the important role of the human gut microbiome and its association with several types of diseases, which explains why the microbiome is called the ‘forgotten organ’^1,2^. In particular, recent studies on type 2 diabetes mellitus (T2DM), a representative disease of metabolic syndrome, have proved the potential that the gut microbiome can be used as an effective tool for diagnosis, treatment, and ultimately prevention of the disease^3^. Seeing that microbiota is found not only in the gut, but also in other parts of the body, including the skin and mouth, and even in the blood, traditionally considered a sterile environment, a better understanding of the dynamics and stability of microbiota will allow us to explain certain diseases with the perspective of external and environmental factors. Consequently, this understanding will help us to illustrate the mechanism of disease more comprehensively. However, so far, except for the gut microbiome, only a few microbiome studies have been attempted for other body sites, and thus the function and mechanism of the other microbiota remain unknown.

Extracellular vesicles (EVs) are spherical particles that are naturally secreted by bacterial and eukaryotic cells into the extracellular environment. EVs were initially thought to only perform the function of removing unwanted materials from the cell, but it has been found that they are also responsible for the mechanism of intercellular or host-bacterial communication. As EVs were known to be able to transfer molecular content to recipient cells and to play an important role in signaling, the effect of EVs on pathological development have gradually been revealed^4^. Therefore, gut microbe-derived EVs have been recently suggested as a useful tool to investigate microbe-host interaction because of stability and penetration of EVs across intestinal barrier compared to microbes^5,6^. Although the stool microbiome is considered as the representative marker of the human microbiome, stool samples provide limited information on microbiome resident in mucosa when compared to direct sampling throughout the human gut^7^. Moreover, the microbial community observed in various body sites have their own characteristics and live adapting to each other. Therefore, identifying the microbiome EVs found across body sites as well as the stool microbiome leads to an understanding of the pathophysiological mechanisms of our body. To the best of our knowledge, there is no study on the correlation analysis of stool microbes and microbe-derived EVs across blood and urine in humans, as well as how differently these results appear between healthy and unhealthy gut barriers in low-grade chronic inflammatory disease, such as T2DM. Most notably, the underlying mechanism involved in insulin resistance and T2DM, leading to a dysregulation of the intestinal barrier, where the alteration of glucagon-like peptide 2 has been reported to increase intestinal permeability often referred to as a ‘leaky gut’, remains unexplored^8^. Meanwhile, the circulating level of lipopolysaccharide, an inflammatory mediator from gut microbiota, also increases in patients with T2DM, although it is unclear whether this dysfunction of intestinal permeability is primarily due to the gut microbiota or T2DM itself^9^.

In this study, we investigated the characteristics of microbial communities of the EVs from serum, urine, and stool samples in Korean adult subjects, and examined the microbial correlation between the sampling sites, which leads to the understanding of the dysfunction of the intestinal barrier in T2DM patients. The barrier assessment is important, but the evaluation of the loss of integrity and function is further hindered by the variability of this functional entity, depending on various factors, including diet. Thus, identifying the association and difference in genomic profiling between the stool microbiome and microbe-derived EVs across the human body can help to evaluate the role of EVs as a biomarker on the intestinal permeability in T2DM.

## Methods

### Ethics approval and consent to participate

The respective Institutional Review Board of Seoul National University reviewed and approved the informed consent and the study protocols and other documents (Permit Number:129792-2015-064). All methods were performed in accordance with the relevant guidelines and regulations.

### Study subjects

Two hundred and ninety-three subjects who had visited Paik Hospital (Seoul, Republic of Korea) for their regular health check from June 2015 to July 2017 were used for the experiment. Samples of their stool, first-void urine, and serum samples after over 8 hours of fasting were collected. The subjects consisted of 99 males and 194 females, with ages ranging from 49 to 87 years. All the subjects reported no current use of antibiotics at the sampling time, though previous antibiotic use relied on the patients’ memory and were minimally collected regarding dosage, duration, and frequency. Among them, 29 cases who had been diagnosed with T2DM were identified. For the sake of an appropriate comparison of comorbidity and demographic factors, 145 matched healthy control were selected among those who did not have any disease case and control groups were matched for age, sex, and BMI.

### EV isolation and DNA extraction from samples

Serum samples were collected into serum separator tubes (SST) and centrifuged at 3,000 rpm for 15 min at 4 °C and mixed with 1x PBS (pH 7.4, Welgene, ML008-01). Stool samples were filtered by a cell strainer after dilution with 10 mL of PBS for 24 hours. After the preparation, samples from serum, stool, and urine were centrifuged at 10,000 × g for 30 min at 4 °C to separate supernatant and pellet. As a result, this sample divided into the pellet contained bacterial cells and the supernatant contained EV. The supernatant was sterilized through a 0.22 μm filter to eliminate bacteria and other foreign particles from sample supernatants. The sterilized supernatants were boiled at 100 °C for 40 minutes to extract DNA from bacterial and EV. Again, remaining floating particles and waste were eliminated by centrifugation at 13,000 rpm at 4 °C for 30 minutes. DNA were extracted using a DNA isolation kit (PowerSoil DNA Isolation Kit, MO BIO, USA) from all sample supernatants plus stool sample pellet according to standard protocol.

### Paired-end reads sequencing

Extracted DNA was amplified with the primer pair, 341F (5′-CCTACGGGNGGCWGCAG-3′) and 805R (5′-GACTACHVGGGTATCTAATCC-3′), to target the hypervariable V3 and V4 regions of the bacterial 16S rRNA gene. DNA libraries were prepared using PCR products after quantification of DNA with a QIAxpert kit (QIAGEN, Germany). The amplicons were pooled at approximately equal molar DNA ratio, and they were sequenced on an Illumina MiSeq (Illumina, USA) according to the manufacturer’s recommendations.

### Analysis of bacterial composition in the microbiota

The barcode and primer sequences in paired-end reads were trimmed by cutadapt version 1.1.6, and the read pair was merged into a single read with CASPER^10,11^. The low-quality reads were discarded with Phred (Q) score-based criteria by Bokulich method, and any reads that did not have a length between 350 and 550 bp were eliminated^12^. After filtering the chimeric sequences, the open-reference Operational Taxonomic Units (OTU) picking method was conducted based on the EzTaxondatabase using UCLUST^13,14^. Reads were clustered into the same OTUs if their sequence similarities were larger than or equal to 97%. After that, representative sequences of OTUs were selected, and singletons were excluded. The representative sequences in each OTU cluster were assigned taxonomy using EzTaxon, and FastTree software in QIIME was used to build the phylogenetic tree^15^. Alpha and beta diversity were calculated with QIIME. The rarefaction curve was obtained with rarefied 1,000 sequences. Unweighted UniFrac was considered for beta diversity and Shannon for alpha diversity.

### Statistical analyses

**Figure 1:**
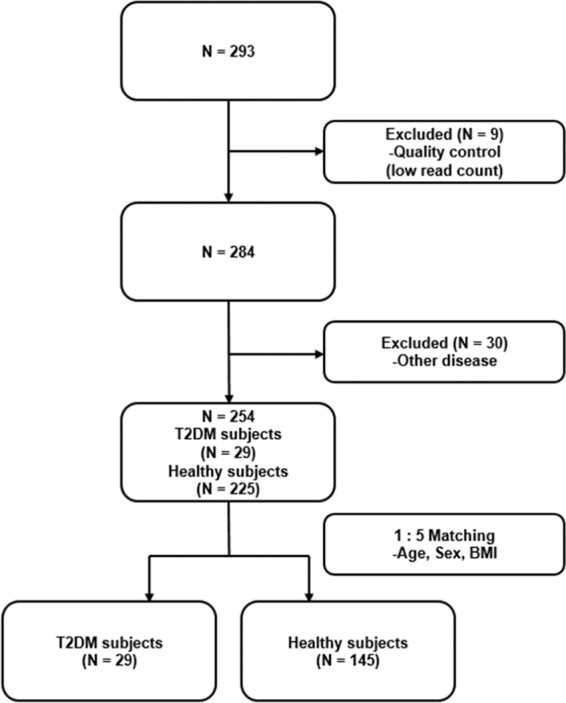
Flowchart of Subjects inclusion/exclusion for correlation analysis.

The samples were collected from a total of 284 subjects consisting of 187 women and 97 men after filtering 9 subjects due to low read count for the subsequent analysis (Fig. 1). After calculating the proportion of subjects with each OTU with more than 1 reads, OTUs with a proportion of less than 5% were excluded^16^ after excluding Archaea and Eukarya. Diabetic patients and healthy subjects matching for age, sex, and BMI were identified using conditional logistic regression. Baseline demographic and clinical characteristics of subjects are reported as mean ± standard deviation for continuous variables and as frequencies and percentages for categorical variables. To identify the correlations among supernatants from different specimens, we first applied Spearman correlations of alpha or beta diversities. Some genera were commonly observed in EVs from different body sites, and, using R for their Pearson correlations, we conducted statistical testing for *H*_0_: r = 0. Microbiome data is highly-skewed and intra- and inter- subject variations tend to be very large. Therefore, permutation-based p-value was obtained from the 100,000 permutation replicates. Permutation p-value is robust for the non-normality and small sample size. Multiple testing problem was adjusted with the false discovery rate (FDR) by the Benjamin Hochberg method^17^. Genera with a correlation coefficient of more than 0.6 and the FDRs of less than 0.05 were considered statistically significant. Lastly, we checked whether correlations shared microbiota are significantly different according to the T2DM status. We performed the likelihood ratio test. For each pair of supernatants from all specimens, we considered their log-transformed relative abundances as response variables, and they were assumed to follow the bivariate normal distributions. T2DM status was included as a covariate, and we assumed that means of log-transformed relative abundance and variances differ by T2DM status. For the reduced model, we assumed the correlations between two groups were the same. Then the likelihood ratio was calculated to test the equivalence of correlations between patients with T2DM and healthy subjects. The permutation p-values were obtained from the 40,000 permuted replicates about the p-value less than 0.05 identified using the chi-square test. All statistical analyses were performed with R Studio (Version 1.1.447.).

## Results

### Comparison of human microbial diversity across the sampling sites including stool, serum, and urine

**Figure 2:**
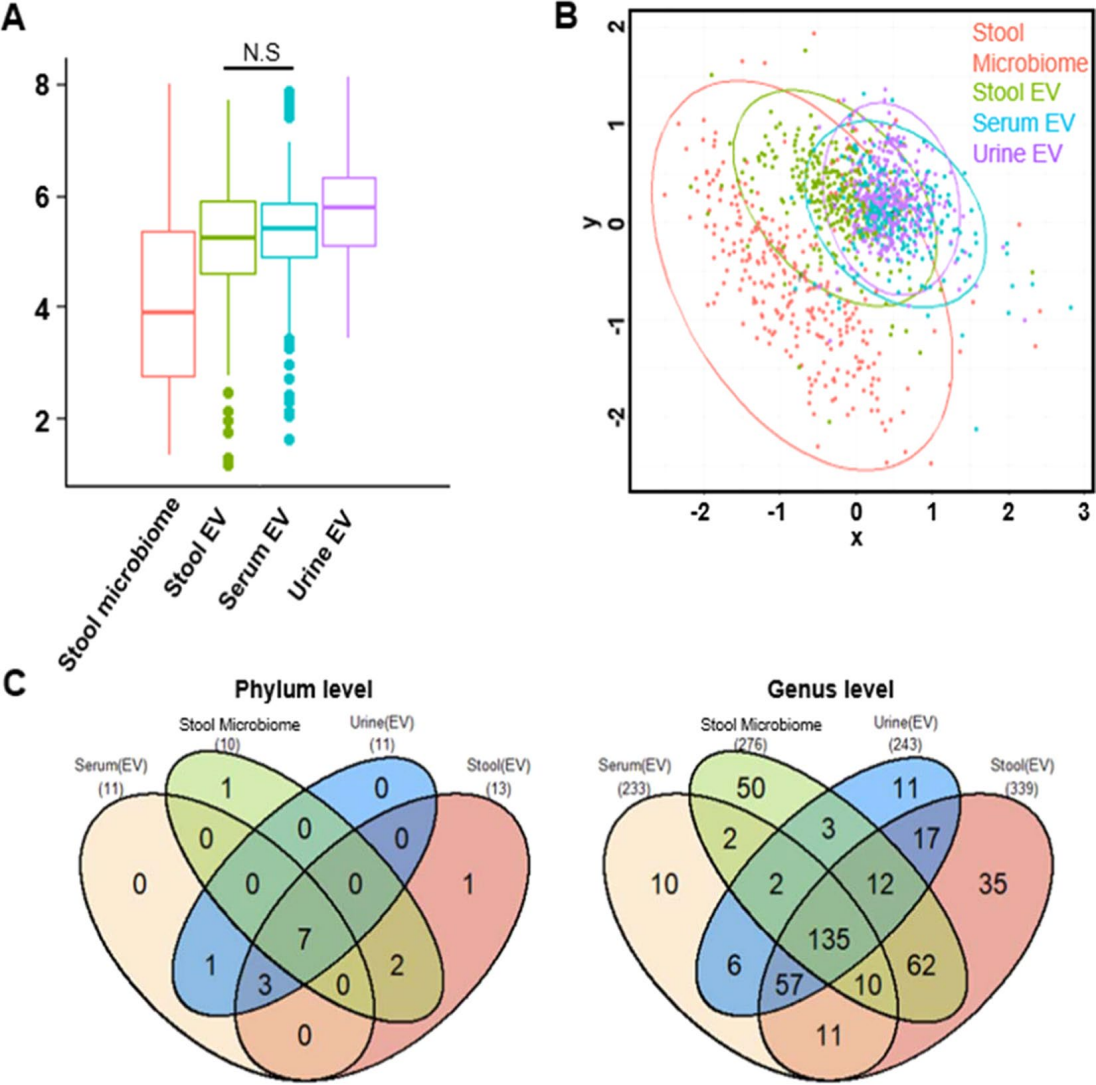
Alpha and Beta Diversity. (**A**) Shannon alpha diversity analyses of the subjects for comparison of body sites. (**B**) NMDS result for beta diversity between body sites. (**C**) The common bacterial genus in both body habitats. The four-way Venn diagram indicates the number of microorganism, and the value in the overlapping circle represents the number of microorganism shared in the site. Phylum level; Genus level.

The mean age of subjects was 63.0 ± 8.5 years, and mean the BMI was 23.7 ± 3.2 kg/*m*^2^ (Table 1). In order to investigate the correlation between various sites including stool, serum, and urine, we initially observed the biodiversity in each site. Alpha diversity differed depending on the sampling sites, except for stool EVs and serum EVs (Fig. 2A). Urine EVs are the most diverse compared to the other EVs (stool microbiome FDR = *4.8* × *10*^*−32*^, serum EVs FDR = *1.0* × *10*^*−5*^, stool EVs FDR = *1.2* × *10*^*−7*^). Non-metric multidimensional scaling (NMDS) results showed the composition of microbiota at the three sites, and EVs data clustered closely and were different from the stool microbiome (Fig. 2B). The composition of subjects from urine and serum EVs was distinct from that of subjects in the stool microbiome, and stool EVs were intermediate. The analysis of the microbiome that exists commonly in all sampling sites revealed these results. Actinobacteria, Bacteroidetes, Firmicutes, Fusobacteria, Proteobacteria, Saccharibacteria_TM7, and Verrucomicrobia were common to all sampling sites at the phylum level (Supplementary Table 1). At the genus level, we identified 233,276,243 and 339 genera from the serum EVs, stool microbiome, urine EVs and stool EVs, respectively, and 145 genera were detected from all sampling sites (Fig. 2C). Stool microbiome and stool EVs had high correlations compared to other sampling sites, representing that stool microbiome and stool EVs are closely clustered (Supplementary Fig. 1). At the genus level, only the genus, *Proteus*, was highly correlated across EV pairs (Supplementary Table 2, serum EVs and stool EVs r = 0.63, serum EVs and urine EVs r = 0.76, urine EVs and stool EVs r = 0.60; FDR = *5.0* × *10*^*−6*^). The genus *Cupriavidus* was significantly correlated between stool microbiome and stool EVs (r = 0.74, FDR = *5.0* × *10*^*−6*^).

**Table 1:** Descriptive statistics of the study participants.

Variables	Reference	All	Case - Control			
			Total subjects	T2DM subjects	Healthy subjects	p-value
Female, N(%)		187 (65.8%)	87 (100%)	13 (14.9%)	74 (85.1%)	0.54
Male, N(%)		97 (34.2%)	87 (100%)	16 (18.4%)	71 (81.6%)	
Age (year)		63.0 ± 8.5	64.0 ± 8.7	64.3 ± 9.7	64.0 ± 8.5	0.90
BMI (m^2^/kg)		23.7 ± 3.2	24.7 ± 3.0	24.7 ± 2.5	24.7 ± 3.1	0.61
Fasting Blood Glucose	70 ~110 mg/dl	100.0 ± 21.2	102.9 ± 24.1	139.2 ± 37.2	95.6 ± 10.5	7.8 × 10^−13^
AST	7 ~ 38 U/L	25.6 ± 11.1	25.9 ± 10.6	25.5 ± 9.9	26.0 ± 10.8	0.69
ALT	4 ~ 43 U/L	24.0 ± 13.6	25.3 ± 13.8	26.7 ± 11.9	25.0 ± 14.2	0.18
γ-GTP	12 ~ 73 IU/L	29.8 ± 30.5	31.3 ± 29.7	30.0 ± 17.8	31.5 ± 31.6	0.30
Creatinine	0.6 ~ 1.2 mg/dl	0.92 ± 0.41	0.93 ± 0.18	0.97 ± 0.24	0.92 ± 0.2	0.28
Total Cholesterol	~ 200 mg/dl	200.0 ± 38.9	194.8 ± 38.8	180.3 ± 37.0	197.7 ± 38.6	0.04
Triglyceride	~ 150 mg/dl	126.2 ± 78.3	127.1 ± 78.1	135.5 ± 75.2	125.4 ± 78.8	0.42
HDL Cholesterol	40 ~ 60 mg/dl	53.0 ± 11.9	50.2 ± 11.1	46.4 ± 9.7	51.0 ± 11.2	0.06
Hemoglobin	14.0 ~ 18.0 g/dl	13.9 ± 1.3	14.1 ± 1.3	13.9 ± 1.3	14.2 ± 1.3	0.76

Descriptive statistics for age and gender of 284 participants and characteristics of diabetic cases and controls. T2DM were selected from cohort and matched in 1:5 ratios with healthy subjects. Factors were sex, age and body mass index (BMI).

BMI: body mass index; AST: aspartate aminotransferase; ALT: alanine aminotransferase; γ-GTP: γ-glutamyltranspeptidase.

### Microbial diversity and its correlation

To find out the biodiversity correlation of the same subjects across the sampling sites, we analyzed the correlation between diversity. The correlation of microbial diversity between sites showed that alpha diversity of EVs pair increased with higher alpha diversity of EVs, though the correlation coefficients were small (Supplementary Fig. 2A, urine EVs and serum EVs; r = 0.15, urine EVs and stool EVs; r = 0.12, stool EVs and serum EVs; r = 0.009). The alpha diversity of stool microbiome showed a relatively high positive correlation with serum, urine, and stool EVs alpha diversity (urine EVs; r = 0.37, stool EVs; r = 0.33, serum EVs; r = 0.24) representing that gut microbiome is a major source of EVs in both serum and urine. On the other hand, beta diversity showed little relationship between the sampling sites compared to alpha diversity. Even though the beta diversity of stool microbiome slightly increased with higher beta diversity of the other EVs (Supplementary Fig. 2B, urine EVs; r = 0.1, stool EVs; r = 0.16, serum EVs; r = 0.009), EVs beta diversity is not correlated with that of other sampling sites.

### The 16S rRNA microbiome correlation between T2DM and healthy subjects

**Figure 3:**
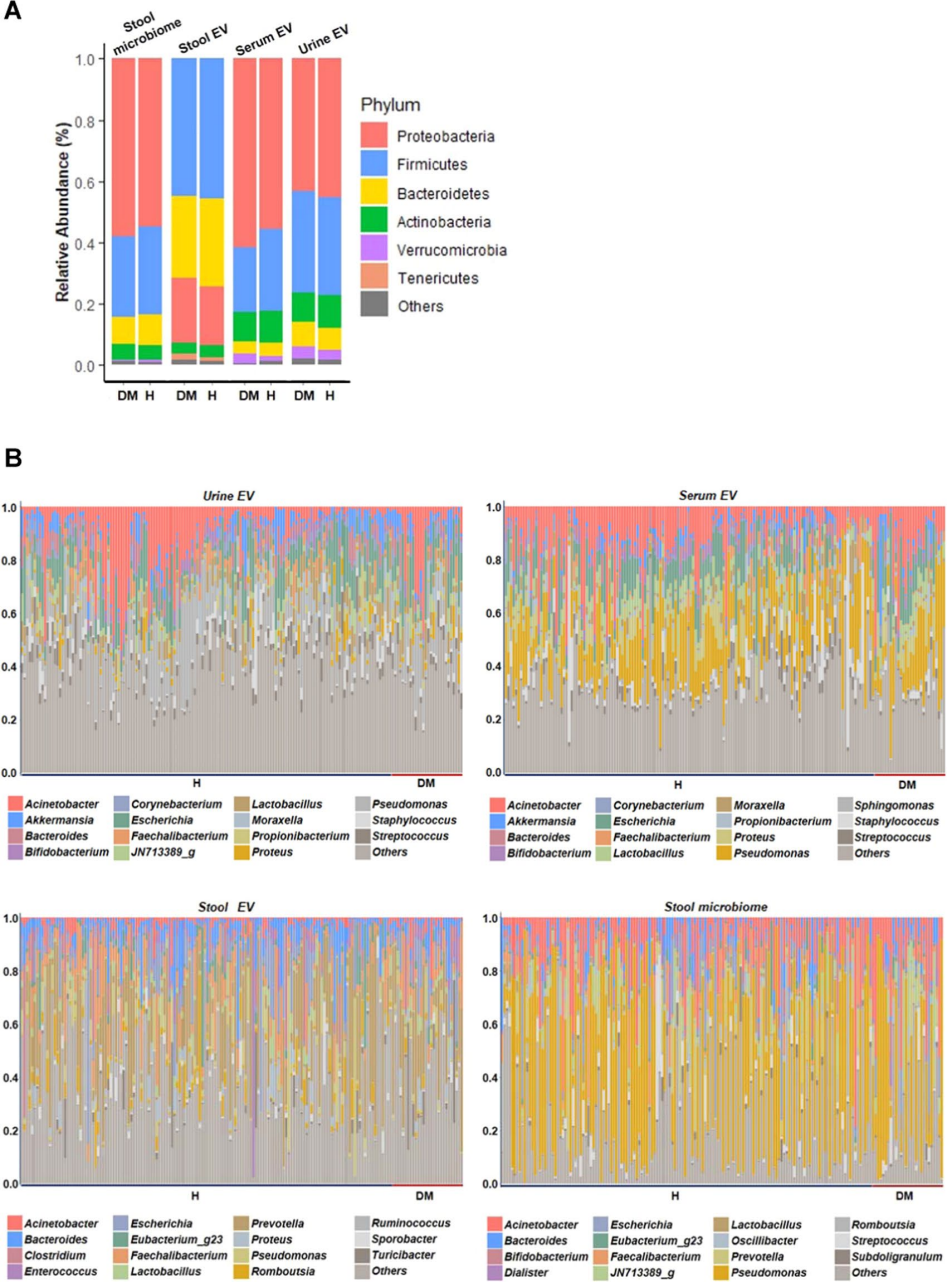
Microbial composition at phylum (Top 5) and genus (Top 15) levels. A: Phylum level, B: Genus level. The bar chart indicates the species present in each body site. The numbers represent the relative proportions of microbiome of people. DM, T2DM patients; H, healthy subjects.

**Figure 4:**
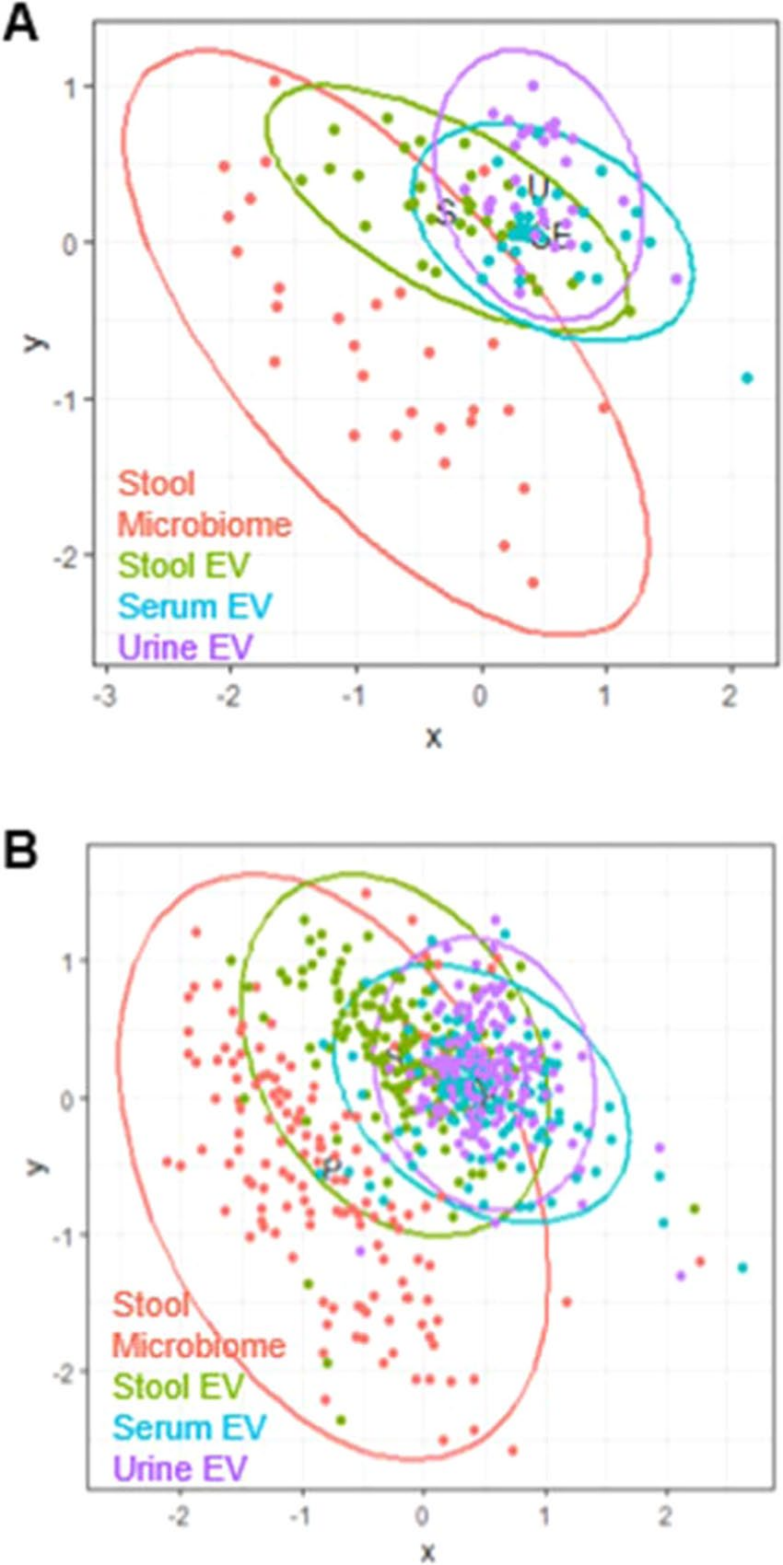
NMDS result for beta diversity between body sites. (**A**) T2DM patients. (**B**) Healthy subjects.

**Figure 5:**
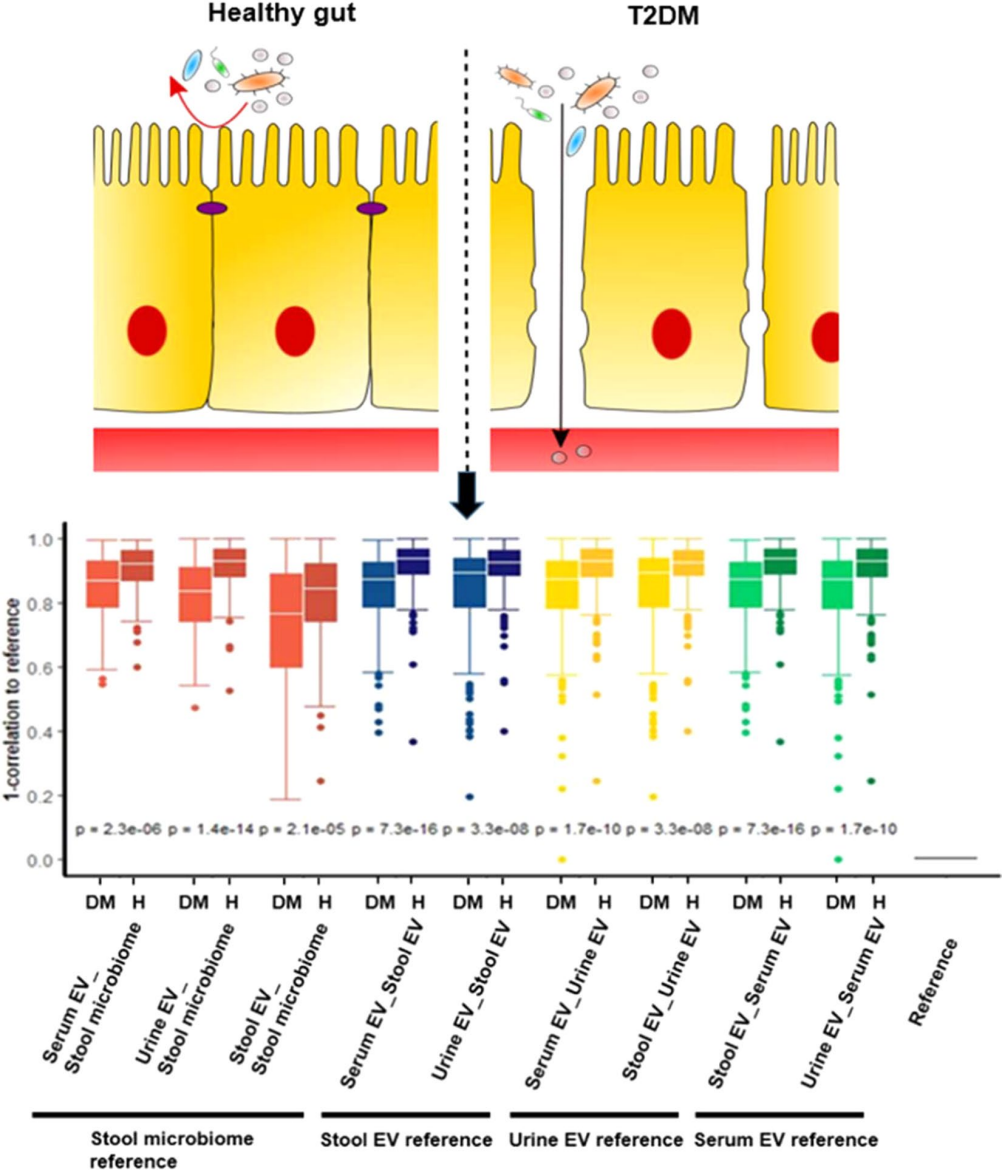
Comparison of correlation coefficients by T2DM status We compared diabetics to healthy subjects and found that the correlation coefficients were higher between body sites in diabetics than those of healthy subjects. We chose all the data once as a reference and then compared the correlations between the groups in relation to the reference. DM indicates the diabetic and H indicates the healthy subjects. P-values indicate the difference between patients with diabetes and healthy subjects. DM, patients with diabetes; H, healthy subjects.

One hundred and seventy-four healthy subjects consisting of 87 women, and 87 men were matched for age, sex, and BMI. The mean age of subjects was 64.0 ± 8.7 years and mean BMI was 24.7 ± 3.0 kg/*m*^2^ (Table 1). Fasting glucose level was significantly high in T2DM subjects compared to the healthy group with p-value of p = *7.8* × *10*^*−13*^ (Table 1). Most of the T2DM subjects were administered with oral hypoglycemic agents (N = 27). Only one subject received insulin monotherapy, and the other received no treatment. In the microbial taxonomic profile, different patterns were observed at each sampling site (Fig. 3). The four most abundant phyla identified were Proteobacteria, Firmicutes, Bacteroidetes and Actinobacteria and the composition of stool EVs was different from the stool microbiome. The microbiome abundance did not significantly differ between T2DM and healthy subjects at the phylum level, except for Firmicutes in serum EVs. (Supplementary Table 3). For both T2DM and healthy subjects, the compositions of EVs were also distinct from that of composition in the stool microbiome, which is consistent with previous findings with a total of 284 subjects (Fig. 4). However, a significantly different correlation across the stool, serum, urine was observed between T2DM and healthy subjects (Fig. 5). With the reference of stool microbiome, subjects with T2DM had much higher correlations compared with healthy subjects, as shown by a serum EVs-stool microbiome, urine EVs-stool microbiome, and stool EVs-stool microbiome (p = *2.3* × *10*^*−6*^, *1.4* × *10*^*−14*^, *2.1* × *10*^*−5*^, respectively). Further analyses with the reference as EVs data also showed consistent results (Fig. 5). T2DM patients, especially in serum EVs reference, showed the biggest difference from healthy subjects (stool-serum p = *7.3* × *10*^*−16*^, urine-serum p = *1.7* × *10*^*−*10^). This means that subjects with T2DM had a substantially higher correlation than healthy subjects across stool, serum, and urine. Further analysis of the correlation between the sites at the genus level showed that 3 genera, *Cupriavidus*, *Escherichia*, and *Proteus*, having more than correlation coefficient 0.6 across sampling sites were associated with T2DM while only one genus, *Proteus*, was associated with healthy subjects (Table 2, Supplementary Fig. 3 FDR < *0.05*). Specifically, the correlations of *Cupriavidus* across stool, serum, and urine were significantly different according to the T2DM status (Table 3, stool EVs and urine EVs p = *2.5* × *10*^*−2*^, serum EVs and urine EVs p = *3.5* × *10*^*−2*^). Therefore, the microbe-associated across body sites is also suggested to be dependent on either T2DM or healthy subjects.

**Table 2:** Significant genus between the sites of T2DM and healthy subjects.

Group	Site	Genus	Coefficient	FDRs p-value
T2DM patients	Serum-Urine	*Cupriavidus*	−0.68	3.2 × 10^−4^
		*Escherichia*	0.62	3.8 × 10^−4^
		*Proteus*	0.78	1.5 × 10^−4^
	Stool-Urine	*Cupriavidus*	0.80	2.5 × 10^−5^
Healthy subjects	Serum-Urine	*Proteus*	0.76	5.0 × 10^−6^
	Stool-Urine	*Proteus*	0.60	5.0 × 10^−6^
	Serum-Stool	*Proteus*	0.63	5.0 × 10^−6^

The correlation of microbiome was assessed with permutation based Pearson correlation. Genera with correlation coefficient more than 0.6 and the FDR less than 0.05 were provided.

**Table 3:** The correlation difference between the sites of T2DM and healthy subjects.

Group	Site	Genus	LR p-value
T2DM patients	Serum-Urine	*Cupriavidus*	0.04
		*Escherichia*	0.34
		*Proteus*	0.77
	Stool-Urine	*Cupriavidus*	0.03
Healthy subjects	Serum-Urine	*Proteus*	0.77
	Stool-Urine	*Proteus*	0.97
	Serum-Stool	*Proteus*	0.81

The correlation difference of significant genus was assessed with a likelihood ratio test.

## Discussion

EVs are already known as one of the communication methods between cells, and they are involved in multiple biological processes by exchanging proteins and genetic materials. Likewise, microbe-derived EVs, which are secreted by bacterial cells, also play crucial roles in host-bacterial communication^4,18^. Recent studies have reported that intestinal microbe-derived EVs can infiltrate the circulatory system through the gut barrier, which is then distributed to the serum, liver, adipose tissue, and skeletal muscle^5,6^. In other words, it indicates that EVs have a possibility of getting involved in the pathogenesis of non-infectious systemic diseases such as T2DM, as well as autoimmune diseases.

Previous human microbiome studies on different body sites have revealed that the sites have a unique composition of the microbial community by characteristics of each habitat, showing strong niche specialization both between- and within-individuals^19,20^. In our research, weak correlations of EV composition across body sites within subjects were also consistent with the previous results. In addition, the other study on microbiome using direct sampling in the gastrointestinal tract showed that the composition and function of the microbiome considerably vary depending on whether the sampling site is in the upper or lower gastrointestinal tract, lumen, or mucus^7^. Therefore, we hypothesized that the healthier the subjects are, the less the EVs between body sites are correlated.

The intestinal barrier is a functional property that selectively limits permeation between gut lumen and mucus or epithelial layer, and its functional status is closely related to commensal microbiota in the gut. The status of the intestinal barrier is newly considered one of the important markers that reveals the pathogenesis of obesity, chronic inflammation, and metabolic syndrome by translocating luminal components into the host^21^. Here, our study revealed that intestinal microbe-derived EVs observed in serum or other tissues could be used for the measure of intestinal permeability and integrity, which indirectly indicates intestinal barrier function. This study confirmed the universal distribution of microbe-derived EVs across body sites, such as stool, serum, and urine, but showed weak correlations of microbial features between the body sites. This means that the composition of EVs among sites within individuals is not only different, but also that, unlike inflammatory bowel disease, the penetrations of EVs across the intestinal barrier rarely occur in the normal population.

Dysregulation of intestinal permeability could be induced by dietary factors and microbiota controlling intestinal inflammation. For instance, high-fat diets alter gut microorganisms, which damage the function of proteins consisting of tight junctions, such as Zonula occlusion^9,22,23^. Additionally, pathological characteristics of obesity and metabolic disorder have been recently explained by the dynamics of the intestinal barrier and microbiota^24,25^. Preclinical and clinical studies clearly showed that the enhancement of intestinal permeability and translocation of bacteria-producing substances, such as endotoxins and EVs, were highly associated with other metabolic diseases as well as T2DM^3,26^. The high concentration of bacterial 16S rDNA in serum could be marker for predicting the onset of T2DM, particularly 6 to 9 years after baseline, more than 90% of which belonged to the Proteobacteria phylum. Moreover, microbe-derived EVs form stool is the key factor in the development of glucose intolerance and insulin resistance, which is the underlying mechanism of T2DM^6^. These findings are consistent with our results, in part because most of serum EVs belong to the Proteobacteria phylum and gram-positive anaerobic bacteria. In the current analysis, the genera of T2DM patients in EV pairs have higher correlation coefficients than healthy subjects across stool, serum, and urine. Four genera were found to be highly correlated and significant in T2DM compared to the control group. The significant genus *Cupriavidus* and *Proteus* are considered opportunistic pathogens associated with human infection in patients with compromised immune systems^27–30^. In addition, genus *Cupriavidus* showed a significant difference between diabetic subjects and healthy subjects, especially in the urine, which might be associated with higher urinary tract infection in T2DM.

Further analysis of representative genus *Akkermansia* and *Oscillibacter* showed positive and negative association with intestinal permeability, respectively, which could also help to understand the difference of intestinal status on permeability between groups^26,31^. Recently, EVs from *Akkermansia muciniphila* reported controlling permeability in the gut, improving the integrity of the intestinal barrier under pathological conditions when high-calorie eating habits were induced^32^. Indeed, the abundances of *Akkermansia* based on our data between T2DM and healthy groups in stools EVs or stool microbiome are 8.95 ± 4.61 vs. 9.26 ± 4.04 or 6.65 ± 3.74 vs. 7.17 ± 4.81, respectively, showing that *Akkermansia* was poor in T2DM group compared to healthy subjects. On the other hand, those of *Oscillibacter* between groups are 12.40 ± 2.37 vs. 11.72 ± 3.08 or 11.47 ± 3.65 vs. 11.00 ± 3.60, respectively, showing that *Oscillibacter* was rich in T2DM. Due to the relatively small sample size in T2DM compared to healthy subjects, the statistically significant difference between groups could not be produced.

The current study has certain limitations. First, the sample size for T2DM was relatively small compared to that of healthy subjects. However, the statistical methods, covariate-adjusted matching, and permutation tests were correctly applied. For the same reason, the genus associated with intestinal permeability was investigated to have no significant difference between groups. Second, 16S rRNA amplicon sequencing after the samples EV only preparation focused on the metagenome profile of EVs, rather than the microbe itself, due to the sterile condition of blood and urine. For this reason, our result of the stool microbiome showed that most bacteria belonged to Proteobacteria phylum, and this was different from that of previous metagenome data in human stool. Third, the assessment of intestinal leakage is only based on the correlation of EVs across body sites. No other intestinal permeability analysis, such as the assessment of epithelial integrity, inflammation biomarkers, and bacterial markers, were performed. Lastly, the causality of the correlation of EVs linked to gut barrier dysfunction in T2DM is hard to know in a pathophysiological viewpoint because our clinical study design was cross-sectional. Despite these limitations, the significant correlation of EVs in T2DM patients showed highly consistent across stool, serum, and urine compared to that of healthy subjects, which could be interpreted as having been due to increased intestinal permeability, as well as lead to frequent urinary tract infection in T2DM. Large-scale and longitudinal studies about stool, blood, and urine EVs need to confirm our findings on the significant correlation of EVs in T2DM, as well as the role of EVs as a biomarker in the intestinal permeability in T2DM.

## Supplementary information

Supplementary materials.
