# Author Correction: Type-2 Diabetics Reduces Spatial Variation of Microbiome Based on Extracellular Vesicles from Gut Microbes across Human Body

**DOI:** 10.1038/s41598-020-62218-1

**Published:** 2020-03-19

**Authors:** Geumkyung Nah, Sang-Cheol Park, Kangjin Kim, Sungmin Kim, Jaehyun Park, Sanghun Lee, Sungho Won

**Affiliations:** 1 Interdisciplinary Program in Bioinformatics, Seoul National University, Seoul, South Korea; 2 Department of public health sciences, Seoul National university, Seoul, South Korea; 3 Inje University College of Medicine and Haeundae Paik Hospital, Busan, South Korea; 4 Department of medical consilience, Graduate school, Dankook university, Yongin-si, South Korea; 5 Institute of Health and Environment, Seoul National University, Seoul, South Korea

Correction to: *Scientific Reports* 10.1038/s41598-019-56662-x, published online 27 December 2019

The original version of this Article contained an error in the title of the paper, where the word “Extracellular” was incorrectly given as “Extracellur”. This has now been corrected in the PDF and HTML versions of the Article and in the accompanying Supplementary Information file.

