# Supplementary material for: Type-2 Diabetics Reduces Spatial Variation of Microbiome Based on Extracellur Vesicles from Gut Microbes across Human Body
Source: Sci Rep. 2019 Dec 27;9:20136. doi: 10.1038/s41598-019-56662-x (PMC6934622; doi:10.1038/s41598-019-56662-x)
Supplement: Supplementary file 1 — Supplementary materials. [file 41598_2019_56662_MOESM1_ESM.docx]

**Supplementary Material for “Type-2 Diabetics Reduces Spatial Variation of Microbiome Based on Extracellur Vesicles from Gut Microbes across Human Body”**

Geumkyung Nah^1^^§^, Sang-Cheol Park^2§^, Kangjin Kim^2^, Sungmin Kym^3^, Jaehyun Park^1^, Sanghun Lee^4^* and Sungho Won^1,2,5^*

^1^Interdisciplinary Program in Bioinformatics, Seoul National University, Seoul, Korea

^2^Department of public health sciences, Seoul National university, Seoul, Korea

^3^Inje University College of Medicine and Haeundae Paik Hospital

^4^Department of medical consilience, Graduate school, Dankook university, Korea

^5^Institute of Health and Environment, Seoul National University, Seoul, Korea.

* Corresponding Authors{Agresti, 2011 #2}:

Sungho Won, Department of Public Health Science, Seoul National University

1 Kwanak-ro Kwanak-gu Seoul 151-742 Korea

(Email) [won1@snu.ac.kr](mailto:won1@snu.ac.kr), (Tel) +82-2-880-2714, (Fax) +82-303-0942-2714

Sanghun Lee, Department of medical consilience, Graduate school, Dankook university

152, Jukjeon-ro, Suji-gu, Yongin-si, Gyeonggi-do, 16890, Korea

(Email) [integrative@korea.com](mailto:integrative@korea.com), (Tel) +82-31-8005-3954

**Supplement Table 1. The common phylum in both body habitats.** The four-way Venn diagram indicates the number of microorganism, and the value in the overlapping circle represents the number of microorganism shared in the site.

| **Site** | **Phylum** |
| --- | --- |
| **Serum EVs - Stool EVs - Urine EVs - Stool microbiome** | **Actinobacteria** |
|  | **Bacteroidetes** |
|  | **Firmicutes** |
|  | **Fusobacteria** |
|  | **Proteobacteria** |
|  | **Saccharibacteria_TM7** |
|  | **Verrucomicrobia** |
| **Serum EVs - Stool EVs - Urine EVs** | **Deinococcus-Thermus** |
|  | **Armatimonadetes** |
|  | **Deferribacteres** |
| **Serum EVs - Urine EVs** | **Acidobacteria** |
| **Stool microbiome - Stool EVs** | **Cyanobacteria** |
|  | **Tenericutes** |
| **Stool microbiome** | **Synergistetes** |
| **Stool EVs** | **Chloroflexi** |

**Supplementary Table 2**. **Significant genus between the sites.**

The correlation of microbiome was assessed with permutation based Pearson correlation. Genera with correlation coefficients more than 0.6 and the FDRs less than 0.05 are provided.

| **Site** | **Genus** | | **Coefficient** | | **FDR** | |  |
| --- | --- | --- | --- | --- | --- | --- | --- |
| **Serum EVs-Stool EVs** | **Proteus** | | 0.63 | | 5.0*${10}^{-6}$ | |  |
| **Serum EVs-Urine EVs** | **Proteus** | | 0.76 | | 5.0*${10}^{-6}$ | |  |
| **Urine EVs-Stool EVs** | **Proteus** | | 0.60 | | 5.0*${10}^{-6}$ | |  |
| **Stool EVs-**  **Stool microbiome** | | **Cupriavidus** | | 0.74 | | 5.0*${10}^{-6}$ | |

**Supplementary Table 3.** **Microbial composition difference.**

Microbial composition difference between T2DM and healthy subjects at phylum (Top 4) levels.

| **Phylum** | **Stool microbiome**  **(FDR)** | **Stool EVs**  **(FDR)** | **Serum EVs**  **(FDR)** | **Urine EVs**  **(FDR)** |
| --- | --- | --- | --- | --- |
| **Proteobacteria** | 0.95 | 0.88 | 0.23 | 0.62 |
| **Firmicutes** | 0.95 | 0.93 | 0.05 | 0.62 |
| **Actinobacteria** | 0.95 | 0.93 | 0.75 | 0.50 |
| **Bacteroidetes** | 0.95 | 0.93 | 0.75 | 0.62 |

| **Supplement Fig 1**. **Comparison of correlation coefficient by body site.** |
| --- |
| The box plot was used to show the correlation between body sites in the genus level. The correlation coefficient(r) has a value between -1 and 1. 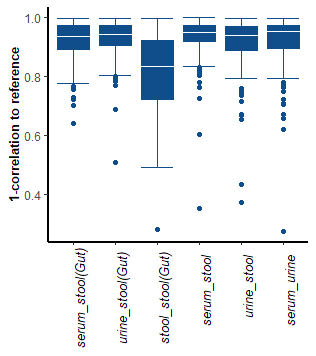 **Serum EV-Stool microbiome**  **Urine EV-Stool microbiome**  **Stool EV-Stool microbiome**  **Serum EV-Stool EV**  **Urine EV-Stool EV**  **Serum EV-Urine EV** |

| **Supplement Fig 2. Biodiversity correlation between various body sites.** |
| --- |
| A scatterplot indicates the relationship between two diversity values measured on the same individuals. The red solid line represents the regression line. A: Alpha diversity, Shannon measure. B: Beta diversity, Unweighted unifrac measure.  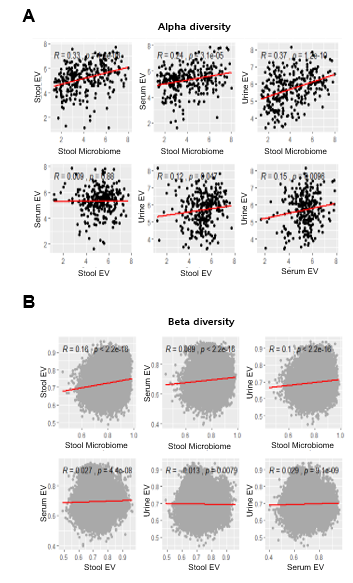 |

| **Supplement Fig 3. Comparison of correlation coefficient by group.** |
| --- |
| Top 20 correlation coefficient difference from each data. Each value is the correlation coefficient in each site. The line is the confidence interval in each site. Thus, values located farther away from the vertical grey zero line indicate a higher correlation coefficient in that genus, values existed closer indicate a lower correlation coefficient. T2DM, diabetic subjects; Healthy, healthy subjects. A: Serum EVs and Stool EVs. B: Serum EVs and Urine EVs. C: Stool EVs and Urine EVs.  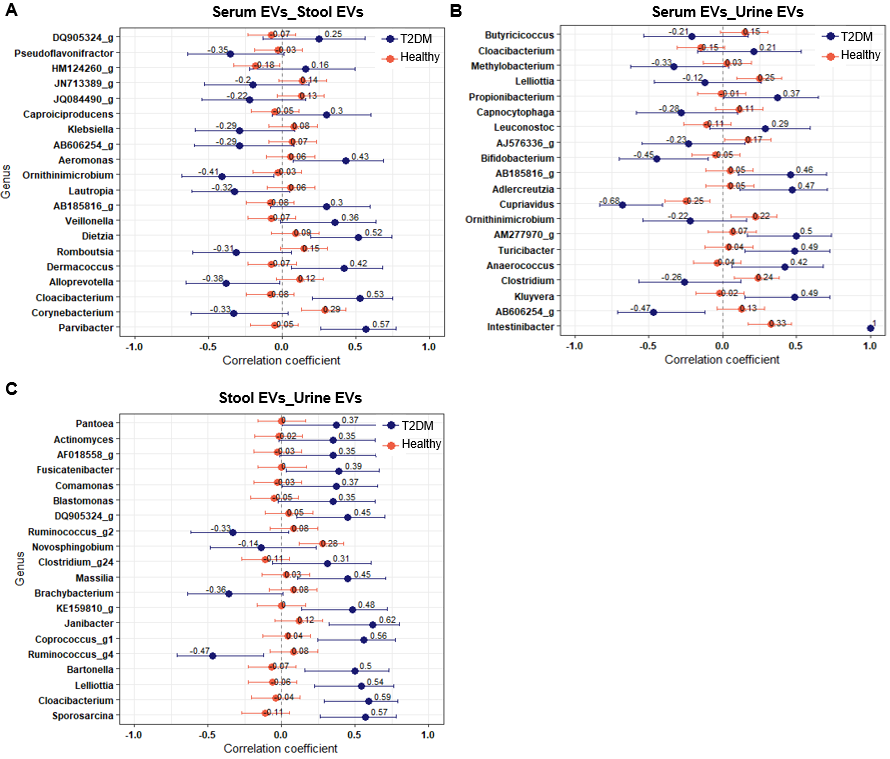 |
